# Ginsenoside Rb1: From basic mechanisms to therapeutic applications in diabetic complications

**DOI:** 10.1016/j.jgr.2026.101070

**Published:** 2026-05-28

**Authors:** Yinli Zhao, Lusha Wei, Junwen Gong, Shuanghua Zheng, Dan Li, Zhiqiang Ke

**Affiliations:** a School of Stomatology and Ophthalmology, Xianning Medical College, Hubei University of Science and Technology, Xianning, Hubei, 437100, China; b School of Basic Medical Sciences, National Demonstration Center for Experimental General Medicine Education, Xianning Medical College, Hubei University of Science and Technology, Xianning, Hubei, 437100, China; c Department of Neonatology, The Clinical Research Center for Acute Myocardial Infarction of Hubei Province, Xianning Central Hospital / First Affiliated Hospital of Hubei University of Science and Technology, Xianning, Hubei, 437100, China; d Hubei Key Laboratory of Industry Microbiology, Hubei University of Technology, Wuhan, 430068, China

**Keywords:** Ginsenoside Rb1, Diabetes mellitus, Insulin resistance, Diabetic complications, Multi-target mechanism

## Abstract

Diabetes is a globally prevalent chronic metabolic disorder characterized by defective insulin secretion and/or insulin resistance, often leading to various severe complications. As a traditional Chinese medicine, *Panax ginseng* has a long-standing history in the adjunctive treatment of diabetes, with ginsenoside Rb1 being one of its most important active constituents. This review systematically summarizes recent advances in basic and clinical research concerning ginsenoside Rb1 in the prevention and treatment of diabetes, with a focus on its pharmacological effects and molecular mechanisms in improving insulin resistance, protecting pancreatic β-cell function, and delaying diabetic complications. Accumulated evidence demonstrates that ginsenoside Rb1 exerts anti-diabetic effects through multi-target and multi-pathway mechanisms, specifically involving the regulation of glucose and lipid metabolism, inhibition of oxidative stress and inflammatory responses, and modulation of autophagy, exhibiting significant therapeutic potential. Based on its favorable pharmacological activities, ginsenoside Rb1 holds promise as a lead compound for diabetes drug development. Future research should further focus on the validation of its molecular targets and the exploration of pathways for clinical translation.

## Graphical abstract

**Figure:**
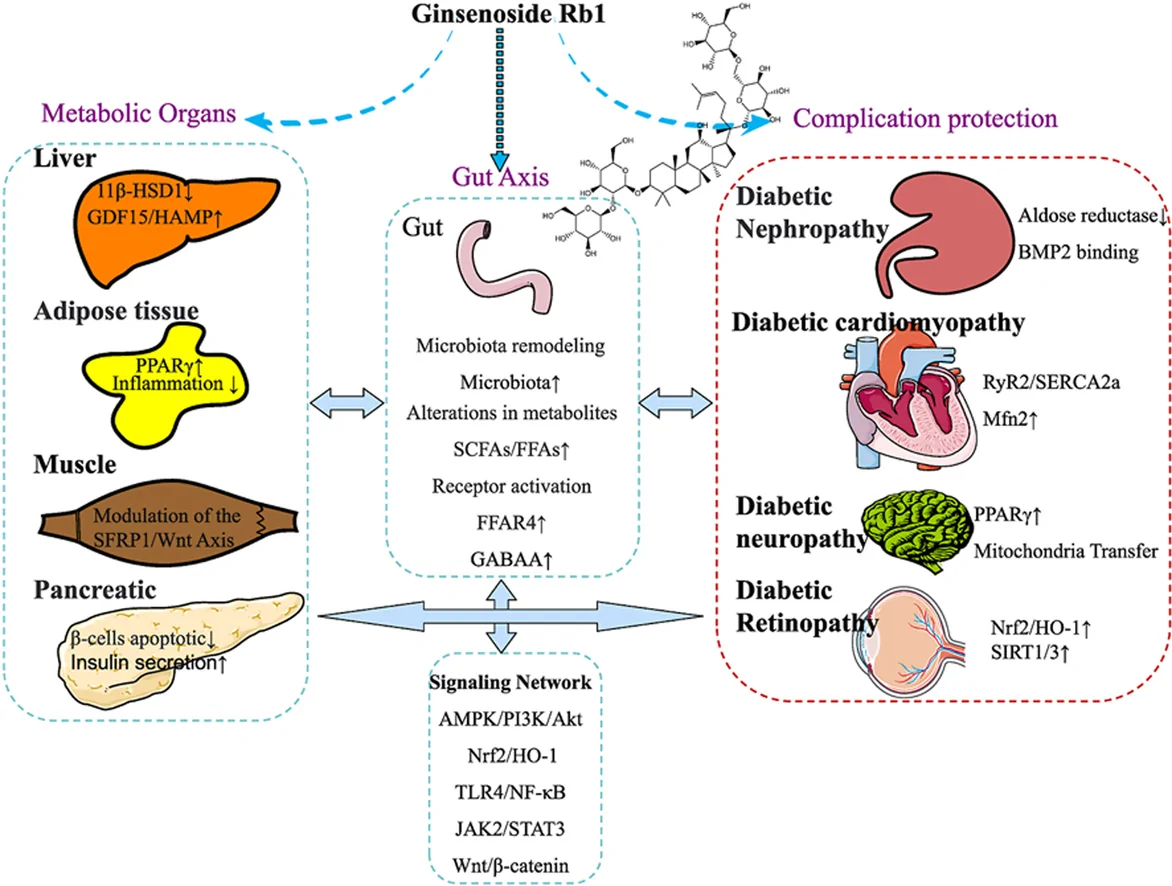

## 1 Introduction

Diabetes mellitus has emerged as one of the most rapidly growing global public health emergencies of the 21st century. It encompasses several categories, with type 2 diabetes mellitus (T2DM) accounting for over 90% of cases [1]. A significant clinical challenge lies in the fact that approximately 40-50% of patients with T2DM develop chronic kidney disease (CKD), leading to a substantial global health burden [2]. This burden is exacerbated by demographic shifts, including population growth and aging, as well as metabolic risk factors. China, bearing the world's largest diabetic population, faces a particularly urgent need to advance precision prevention and management strategies [1]. Given the limitations of current monotherapeutic approaches, there is growing interest in botanical drugs with multi-target mechanisms. *Panax quinquefolius* (American ginseng) and its close relative *Panax ginseng* have demonstrated hypoglycemic effects through mechanisms such as reversible inhibition of α-amylase and amelioration of hepatic insulin resistance [3]. The primary bioactive constituents of these plants are ginsenosides, a family of triterpene saponins. Among these, ginsenoside Rb1 stands out as one of the most extensively studied and representative compounds.

**Fig. 1:**
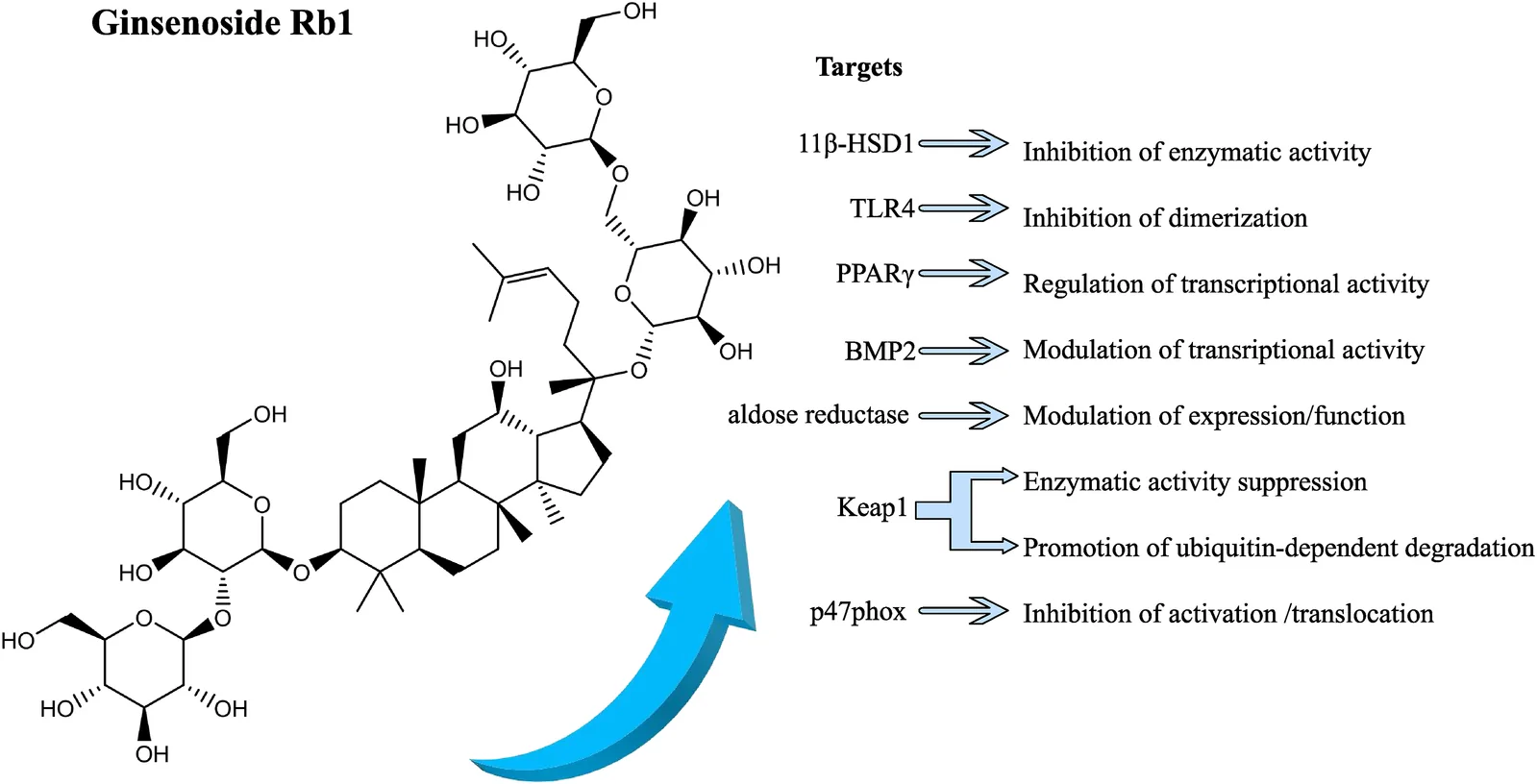
Chemical structure of ginsenoside Rb1 and its key molecular targets. Ginsenoside Rb1 exerts multiple pharmacological effects via direct binding to and regulation of key molecular targets, including 11β-hydroxysteroid dehydrogenase type 1 (11β-HSD1), Toll-like receptor 4 (TLR4), peroxisome proliferator-activated receptor gamma (PPARγ), bone morphogenetic protein 2 (BMP2), aldose reductase (AR), Kelch-like ECH-associated protein 1 (Keap1), and p47phox (a cytosolic subunit of NADPH oxidase). Arrows indicate the specific regulatory outcomes of ginsenoside Rb1 on each target, such as inhibition of enzymatic activity, suppression of protein dimerization, promotion of ubiquitin-dependent degradation, and modulation of transcriptional activity.

While a substantial body of experimental evidence suggests the therapeutic potential of ginsenoside Rb1 in diabetes, a comprehensive synthesis of its mechanisms of action, particularly in the context of diabetic complications like nephropathy, remains fragmented. Therefore, this review aims to systematically evaluate the experimental and clinical evidence concerning the role of ginsenoside Rb1 in the prevention and treatment of diabetes and its associated complications. We will delve into its molecular mechanisms, critically assess the gap between preclinical promise and clinical application, and propose directions for future research to facilitate the translation of this natural product into viable therapeutic strategies. The chemical structure of ginsenoside Rb1 and its validated direct molecular targets are illustrated in [Fig. 1]. Obesity and depression are included in this review as relevant comorbidities of diabetes, given their shared pathophysiological links and the potential of ginsenoside Rb1 to address these interconnected conditions.

## 2 Mechanisms of ginsenoside Rb1 in improving glucose and lipid metabolism and insulin resistance

### 2.1 Enhancement of peripheral insulin sensitivity

Ginsenoside Rb1 improves peripheral insulin sensitivity through coordinated actions across multiple organs and signaling pathways. A recent study demonstrated that ginsenoside Rb1 ameliorates insulin resistance in high-fat diet-induced type 2 diabetic mice by reducing fasting blood glucose, improving glucose tolerance, and increasing insulin sensitivity. Mechanistic investigations revealed that ginsenoside Rb1 suppresses 11β-hydroxysteroid dehydrogenase type I (11β-HSD1) expression in liver and adipose tissue, and overexpression of 11β-HSD1 abolished these protective effects, identifying this enzyme as a key molecular target for its anti-diabetic activity [4]. 11β-HSD1 catalyzes the conversion of inactive cortisone to active cortisol in liver and adipose tissue; elevated cortisol levels promote gluconeogenesis, adipogenesis, and insulin resistance. Thus, repression of 11β-HSD1 by ginsenoside Rb1 reduces local cortisol production, thereby ameliorating hepatic glucose output and adipose tissue dysfunction. In skeletal muscle, the mechanisms underlying ginsenoside Rb1 action may involve modulation of developmental and metabolic signaling pathways. Although not conducted in the context of diabetes, a study by Yin et al. in a tendinopathy model demonstrated that ginsenoside Rb1 upregulated SFRP1 expression, leading to inhibition of excessive Wnt/β-catenin signaling activation, which in turn improved mitochondrial function and calcium homeostasis while reducing ectopic fat deposition and fibrosis [5]. Given that aberrant Wnt signaling activation and mitochondrial dysfunction represent central mechanisms driving insulin resistance and pathological alterations in diabetic skeletal muscle—including ectopic lipid deposition and functional decline—the ginsenoside Rb1-SFRP1-Wnt-mitochondrial function axis identified in this study provides indirect evidence supporting a potential role for ginsenoside Rb1 in ameliorating diabetes-related myopathy and systemic glucose metabolism.

In the liver, a central organ governing glucose and lipid metabolism, ginsenoside Rb1 demonstrates capacity for global metabolic regulation through modulation of systemic factors. Using spatial transcriptomics, Guo et al. found that a specific combination of ginsenoside Rb1 and berberine remodeled the metabolic gene expression network across the hepatic lobule through synergistic regulation of the growth differentiation factor 15/hepcidin axis [6]. This study revealed, at spatial resolution, the capacity of natural product combinations to optimize hepatic metabolic zonation and established the GDF15/hepcidin axis as a novel synergistic target, providing a foundation for developing multi-target diabetes therapies aimed at restoring hepatic metabolic homeostasis. In adipose tissue, a separate investigation demonstrated that ginsenoside Rb1 attenuates tissue inflammation and insulin resistance by regulating macrophage activation through PPARγ. Network pharmacology and molecular docking identified PPARγ as a direct target of ginsenoside Rb1, with subsequent experiments confirming that ginsenoside Rb1 upregulated PPARγ expression, blocked NF-κB activation, and reduced pro-inflammatory cytokine production while promoting IL-10 expression in a co-culture model of adipose-derived stem cells and primary macrophages. These findings suggest that ginsenoside Rb1 may alleviate early-stage insulin resistance in type 2 diabetes through PPARγ-mediated modulation of macrophage polarization and inflammatory responses [7]. Collectively, these studies illustrate that ginsenoside Rb1 enhances peripheral insulin sensitivity through distinct yet complementary mechanisms in different metabolic tissues—modulating hepatic metabolic zonation via the GDF15/hepcidin axis while attenuating adipose tissue inflammation through PPARγ-dependent macrophage regulation.

### 2.2 Modulation of gut microbiota and short-chain fatty acid metabolism

Several recent investigations have elucidated the role of gut microbiota in mediating the metabolic effects of ginsenoside Rb1. In a study using high-fat diet-induced Kkay mice, a model of type 2 diabetes, ginsenoside Rb1 administration improved glucose tolerance, reduced insulin resistance, and attenuated pancreatic and hepatic injury. These improvements were associated with modulation of gut microbiota composition and alterations in fecal free fatty acid metabolites. Notably, the anti-diabetic effects of ginsenoside Rb1 were abolished following antibiotic treatment, demonstrating that its mechanism of action is dependent on the gut microbiota [8]. A separate study examined the effects of ginsenoside Rb1, salvianolic acid B, and their combination in high-fat diet-induced obese mice. Following eight weeks of intervention, all treatments significantly improved fasting blood glucose, glycated hemoglobin, lipid profiles, and glucose tolerance, while both ginsenoside Rb1 alone and the combination reduced body weight. Analysis of gut microbiota revealed that each treatment altered microbial composition and reduced overall diversity, with specific taxonomic changes associated with each intervention, suggesting that the metabolic improvements may be partially mediated through modulation of the gut microbiota [9]. Although ginsenoside Rb1 exhibits low oral bioavailability, increasing evidence supports a “gut-axis” mechanism whereby it exerts systemic metabolic regulatory effects through direct action on the intestine and remodeling of the gut microbiota ecosystem. A study by Zou et al. provided direct evidence for this mechanism in a high-fat diet-induced metabolic disorder model. Ginsenoside Rb1 selectively enriched beneficial bacteria while inhibiting harmful populations, which subsequently altered the metabolic profile of long-chain fatty acids and activated the downstream receptor FFAR4 (free fatty acid receptor 4). This “microbiota-metabolite-receptor” regulatory cascade led to significant improvements in systemic glucose and lipid metabolism disorders [10]. Mechanistically, this finding offers a plausible explanation for the notable pharmacological activity of Ginsenoside Rb1 despite its low bioavailability, suggesting that its systemic benefits originate from localized effects within the intestinal tract. Therapeutically, the identification of FFAR4 as a key metabolic sensor—whose activation is closely associated with improved insulin sensitivity and enhanced glucagon-like peptide-1 secretion—positions the “gut microbiota-FFAR4” axis as a promising target for developing novel modulators for metabolic diseases such as type 2 diabetes and hyperlipidemia, representing an approach distinct from conventional direct glucose-lowering or lipid-lowering drugs. Collectively, these studies establish that ginsenoside Rb1 improves glucose and lipid metabolism through gut microbiota-dependent mechanisms, involving modulation of microbial composition, alteration of metabolite profiles, and activation of host receptors such as FFAR4, thereby providing a mechanistic basis for its therapeutic effects despite limited systemic bioavailability.

This study investigated the role of gut microbiota in mediating the neuroprotective effects of ginsenoside Rb1, addressing the apparent contradiction between its low brain exposure and pharmacological activity. Using a pseudo germ-free rat model of cerebral ischemia/reperfusion injury, the researchers demonstrated that the neuroprotective effects of ginsenoside Rb1 were microbiota-dependent, with ginsenoside Rb1 pretreatment significantly increasing the relative abundance of *Lactobacillus helveticus* by 15.26-fold. Direct colonization with *L. helveticus* reproduced the protective effects of ginsenoside Rb1 against ischemic injury and inflammatory cytokine production, while both ginsenoside Rb1 and *L. helveticus* upregulated GABA receptor expression—effects that were attenuated by a GABAA receptor antagonist, suggesting that ginsenoside Rb1 exerts neuroprotection through microbiota-mediated modulation of GABAergic signaling [11]. These findings elucidate a novel mechanism by which ginsenoside Rb1 exerts central nervous system effects through peripheral modulation of gut microbiota, reconciling the apparent discrepancy between its limited brain penetration and potent neuroprotective activity, and positioning the ginsenoside Rb1-microbiota-GABA receptor axis as a potential therapeutic target for ischemic stroke.

### 2.3 Antioxidant effects

To systematically delineate the multifaceted antioxidant mechanisms of Ginsenoside Rb1 [Table 1], summarizes its reported molecular targets, upstream regulatory events, downstream signaling pathways, and corresponding functional outcomes across various cell and tissue types. A recent evaluation of four processing methods for American ginseng revealed that vacuum freeze-drying best preserved the microstructure and color of fresh ginseng. Low-temperature softening followed by hot air drying yielded the highest contents of ginsenosides Rg1 and ginsenoside Rb1, while steaming followed by hot air drying generated rare ginsenosides including Rg6, Rg3 isomers, Rk1, and Rg5, accompanied by enhanced antioxidant activity. Principal component analysis based on ginsenoside and volatile compound profiles distinguished the processed products and identified potential bioactive components associated with color development and antioxidant properties [19]. Separate mechanistic investigations have elucidated the antioxidant actions of ginsenoside Rb1 at the cellular level. In human umbilical vein endothelial cells exposed to hydrogen peroxide, ginsenoside Rb1 activated AMPK phosphorylation and restored SIRT1 expression, leading to increased endothelial nitric oxide synthase expression and nitric oxide production alongside suppressed PAI-1 expression. Inhibitor studies confirmed that both AMPK and SIRT1 mediate ginsenoside Rb1 protection against oxidative stress-induced cellular senescence [12]. An additional study developed biomimetic nanoparticles through co-assembly of ginsenoside Rb1 and probucol without excipients, subsequently coated with macrophage-derived microvesicles for targeted atherosclerosis therapy. Following intravenous administration in a mouse atherosclerosis model, these nanoparticles accumulated at plaque sites and attenuated disease progression through combined reduction of oxidative stress, suppression of inflammatory mediators, and inhibition of lipid deposition. Long-term treatment with this formulation produced no observable adverse effects [20]. Collectively, these findings from processing optimization, mechanistic cellular studies, and advanced drug delivery approaches provide multi-level evidence supporting the therapeutic potential of ginsenoside Rb1 as an antioxidant agent.

**Table 1:** Comparison of antioxidant mechanisms of ginsenoside Rb1.

Direct Molecular Target	Upstream Events	Downstream Signaling Pathways	Cell/Tissue Type	Functional Effects	Reference
Keap1 (direct binding)	SYVN1-mediated degradation ↑, Keap1 protein level ↓	Nrf2 nuclear translocation ↑, PGC-1α ↑, HO-1 ↑, GCLC ↑, GCLM ↑	Vascular endothelial cells, cardiomyocytes	Antioxidant enzyme expression ↑	[[12], [13], [14]]
p47phox (direct binding)	Membrane translocation ↓	NOX2 activity ↓, O_2_^−^ production ↓	Vascular cells	ROS production ↓	[13]
Mitochondrial Complex I (ND3 subunit)	ND3 subunit binding, deactive conformation maintained	NADH dehydrogenase activity ↓	Cardiomyocytes, astrocytes	Mitochondrial ROS production ↓	[15,16]
SIRT1/3 (indirect)	NAD+/NADH balance regulation	SIRT activity ↑, eNOS ↑, NO ↑	Retinal endothelial cells	mtDNA copy number ↑, mitochondrial function ↑, cellular senescence ↓	[12,17]
AMPK (indirect)	Phosphorylation activation	SIRT1 ↑, eNOS ↑, PAI-1 ↓	Endothelial cells	Oxidative stress-induced senescence ↓	[12]
Nrf2 (indirect)	Nuclear translocation ↑	GCLC ↑, GCLM ↑, HO-1 ↑	Retina, cardiomyocytes	Antioxidant capacity ↑, oxidative stress ↓	[14,18]

***Abbreviation***: AMPK, AMP-activated protein kinase; eNOS, endothelial nitric oxide synthase; GCLC, glutamate-cysteine ligase catalytic subunit; GCLM, glutamate-cysteine ligase modifier subunit; HO-1, heme oxygenase 1; Keap1, Kelch-like ECH-associated protein 1; NAD+/NADH, nicotinamide adenine dinucleotide (oxidized/reduced); NOX2, NADPH oxidase 2; Nrf2, nuclear factor erythroid 2-related factor 2; p47phox, a cytosolic subunit of NADPH oxidase; PGC-1α, peroxisome proliferator-activated receptor gamma coactivator 1-alpha; SIRT, sirtuin; SYVN1, synoviolin 1 (an E3 ubiquitin ligase).

### 2.4 Anti-inflammatory effects

[Table 2] summarizes the diverse anti-inflammatory mechanisms of ginsenoside Rb1 as reported in various cellular and tissue models. Mechanistic investigations have demonstrated that ginsenoside Rb1 exerts anti-inflammatory effects through direct binding to TLR4, which prevents receptor dimerization and subsequently inhibits the TLR4-MyD88-NF-κB/MAPK signaling cascades, leading to reduced pro-inflammatory cytokine release. In vivo studies confirmed that ginsenoside Rb1 attenuates acute kidney injury, protects against LPS-induced septic mortality, and reduces ear edema in mouse models, establishing TLR4 as a direct molecular target mediating its anti-inflammatory activity [21]. A related study reported the enzymatic production of gypenoside XVII from ginsenoside Rb1 using a recombinant β-glucosidase derived from *Bifidobacterium dentium*, achieving complete conversion with 100% molar yield under optimized conditions. Pharmacological evaluation revealed that gypenoside XVII exhibits enhanced anti-inflammatory activity compared to its precursor ginsenoside Rb1, demonstrating greater inhibition of TNF-α and IL-6 production in LPS-stimulated macrophages and superior suppression of xylene-induced ear edema in mice. This enzymatic approach enables large-scale production of gypenoside XVII, which represents a promising anti-inflammatory candidate with improved potency [25]. Separately, researchers developed mannose-modified bovine serum albumin nanoparticles (Man-BSA@Rb1) to address the solubility limitations and lack of targeted delivery associated with ginsenoside Rb1. This formulation enhanced cellular uptake and anti-inflammatory effects in LPS-stimulated macrophages through inhibition of NF-κB and MAPK signaling pathways, while also demonstrating therapeutic efficacy in a d-Gal/LPS-induced mouse model of liver injury. The Man-BSA delivery system offers an effective strategy for improving the therapeutic application of ginsenoside Rb1 in inflammatory conditions [26]. Collectively, these investigations at the molecular target identification, structural optimization, and formulation development levels provide complementary strategies for harnessing and enhancing the anti-inflammatory potential of ginsenoside Rb1.

**Table 2:** Summary of anti-inflammatory mechanisms of ginsenoside Rb1 in different cell/tissue models.

Direct Molecular Target	Upstream Events	Downstream Signaling Pathways	Cell/Tissue Type	Functional Effects	Reference
TLR4 (direct binding)	TLR4 dimerization ↓	MyD88/NF-κB ↓, MAPK ↓	Macrophages, kidney	Pro-inflammatory cytokines ↓ (TNF-α, IL-6), ear edema ↓, sepsis mortality ↓	[21]
PPARγ (direct binding)	PPARγ expression ↑	NF-κB nuclear translocation ↓	Adipose tissue macrophages	IL-10 ↑, M2 polarization ↑, tissue inflammation ↓	[7]
NLRP3 (indirect)	Mitophagy ↑	ROS production ↓	Astrocytes	Pyroptosis ↓	[22]
TLR4/NF-κB/C3	C3 production ↓	Microglial activation ↓, synaptic elimination ↓	Hippocampal astrocytes	Depressive behavior ↓, synaptic structure preserved	[23]
MAPK/NF-κB	Glucocorticoid receptor function restored	IDO activity ↓, 5-HT ↑, 5-HT1A receptor ↑	Brain, hippocampus	Neuroinflammation ↓, antidepressant effect	[24]

***Abbreviation***: TLR4, toll-like receptor 4; MyD88, myeloid differentiation primary response 88; NF-κB, nuclear factor kappa-light-chain-enhancer of activated B cells; MAPK, mitogen-activated protein kinase; TNF-α, tumor necrosis factor alpha; IL-6, interleukin 6; PPARγ, peroxisome proliferator-activated receptor gamma; IL-10, interleukin 10; NLRP3, NLR family pyrin domain containing 3; ROS, reactive oxygen species; C3, complement component 3; IDO, indoleamine 2,3-dioxygenase; 5-HT, serotonin; 5-HT1A, serotonin receptor 1A.

Note: The table summarizes findings from multiple studies; arrows indicate upregulation (↑) or downregulation (↓). References are listed in brackets.

**Fig. 2:**
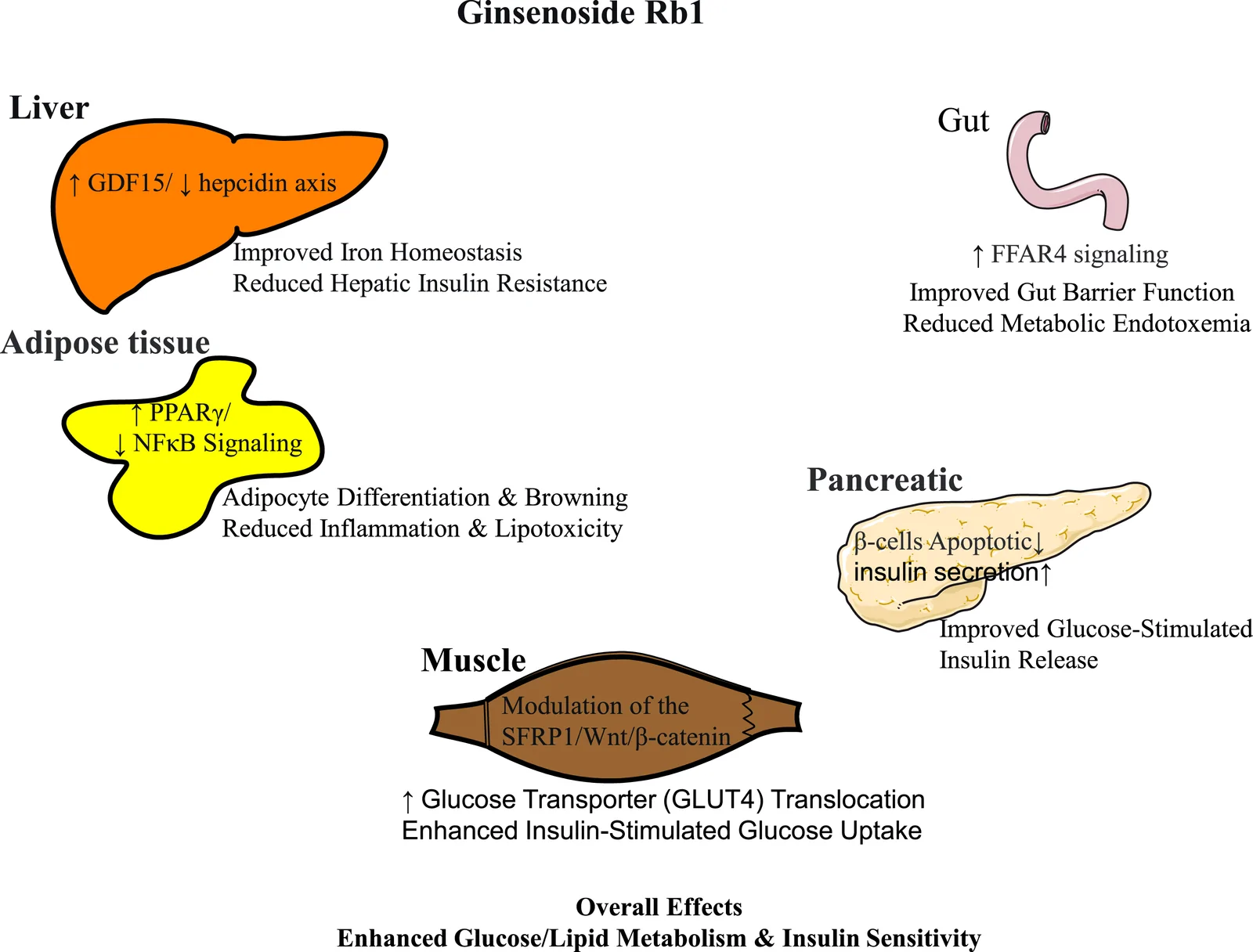
Tissue-specific actions and inter-organ cross-talk of ginsenoside Rb1 in improving glucose/lipid metabolism and insulin resistance. Ginsenoside Rb1 exerts coordinated effects across multiple metabolic organs and the gut ecosystem. In the liver, it regulates the GDF15/hepcidin axis to optimize metabolic zonation. In adipose tissue, it activates PPARγ to suppress NF-κB-mediated inflammation and improve insulin sensitivity. In skeletal muscle, it modulates the SFRP1/Wnt/β-catenin pathway, enhancing mitochondrial function and reducing ectopic lipid deposition. In the intestine, ginsenoside Rb1 remodels gut microbiota composition and activates FFAR4 signaling, which contributes to systemic metabolic benefits. These tissue-specific actions are interconnected through organ crosstalk, collectively ameliorating insulin resistance and restoring glucose/lipid homeostasis.

[Table 3] integrates the representative molecular axes through which Ginsenoside Rb1 exerts dual anti-inflammatory and antioxidant effects via dual-target or multi-target regulation. In contrast to the preceding sections that focused on individual effects, this table centers on how a single molecular event can concurrently mediate both antioxidant and anti-inflammatory responses, aiming to elucidate its “one target, multiple effects” or “multi-axis synergistic” mode of action. The tissue-specific actions and inter-organ cross-talk of ginsenoside Rb1 in improving glucose/lipid metabolism and insulin resistance are summarized in [Fig. 2].

**Table 3:** Comparison of dual anti-inflammatory and antioxidant mechanisms of ginsenoside Rb1.

Direct Molecular Target	Upstream Events	Downstream Signaling Pathways	Cell/Tissue Type	Functional Effects	Reference
AMPK/Nrf2/NF-κB network	AMPK phosphorylation ↑	Nrf2 ↑, NF-κB ↓	Multiple cell types	Metabolic regulation + antioxidant + anti-inflammatory	[12,14,18]
Nrf2/PGC-1α/NOX2 axis	Keap1 degradation ↑, p47phox translocation ↓	Nrf2/PGC-1α ↑, NOX2 ↓	Vascular cells	Endogenous antioxidant defense ↑, pro-oxidant enzyme activity ↓	[13]
GDF15/Hepcidin axis	Synergistic regulation	Hepatic metabolic zonation remodeling	Liver	Metabolic homeostasis restoration, oxidative stress ↓	[6]

***Abbreviation***: AMPK, AMP-activated protein kinase; GDF15, growth differentiation factor 15; Keap1, Kelch-like ECH-associated protein 1; NF-κB, nuclear factor kappa-light-chain-enhancer of activated B cells; NOX2, NADPH oxidase 2; Nrf2, nuclear factor erythroid 2-related factor 2; p47phox, neutrophil cytosolic factor 1 (a subunit of NOX2); PGC-1α, peroxisome proliferator-activated receptor gamma coactivator 1-alpha. Arrows (↑ and ↓) denote an increase or decrease in expression or activity, respectively.

## 3 Protective effects of ginsenoside Rb1 on pancreatic β-cells

Maintaining the mass and function of functional pancreatic β-cells is central to correcting glucose homeostasis dysregulation in diabetes. Ginsenoside Rb1 not only directly enhances the insulin secretory function of β-cells but also improves their defense and survival capabilities under metabolic stress conditions through multifaceted mechanisms, thereby achieving comprehensive protection of β-cells.

### 3.1 Promotion of insulin secretion

Ginsenoside Rb1 can directly act on pancreatic β-cells, exerting incretin-mimetic effects. A study by Park et al. demonstrated that ginsenoside Rb1, in conjunction with its congener Rg1, synergistically activates key PKA/cAMP and PI3K/Akt signaling pathways within β-cells [27]. This dual activation pattern, resembling the action profile of glucagon-like peptide-1 (GLP-1) receptor agonists, effectively enhances glucose-stimulated insulin secretion, revealing at the molecular level the direct mechanism by which ginsenoside Rb1 improves β-cell secretory function. A separate investigation into the anti-hyperlipidemic effects of ginseng total saponins (GTS) in high-fat diet-fed rats provided additional insights into insulinotropic actions of ginsenoside Rb1. GTS treatment for four weeks ameliorated hyperlipidemia, reduced body fat and liver weight, and improved insulin resistance, accompanied by increased portal GLP-1 levels, enhanced intestinal GLP-1 content, and elevated L-cell numbers. In vitro experiments using NCI-H716 cells further confirmed that both GTS and ginsenoside Rb1 stimulated GLP-1 secretion, suggesting that the metabolic improvements following GTS treatment may be mediated in part through enhanced GLP-1 release [28]. Collectively, these findings indicate that ginsenoside Rb1 promotes insulin secretion through complementary mechanisms involving both direct activation of intracellular signaling pathways in pancreatic β-cells and indirect stimulation of intestinal GLP-1 release, thereby exerting dual incretin-enhancing effects.

### 3.2 Inhibition of β-cell apoptosis and attenuation of glucolipotoxicity

Apoptosis induced by a high-glucose and high-lipid environment is a key factor in the progressive loss of pancreatic β-cells. Ginsenoside Rb1 exerts a definitive protective effect against this process. Studies have shown that in β-cell apoptosis models induced by high glucose or inflammatory factors, treatment with ginsenoside Rb1 significantly reduces the apoptotic rate. The underlying mechanism involves downregulating the expression of pro-apoptotic proteins Bax and Fas, as well as inhibiting the activation of the executioner caspase-3 [29]. Furthermore, the protective effects of ginsenoside Rb1 are associated with its modulation of a broad cellular stress network. As reviewed by Zhou et al., ginsenoside Rb1 helps β-cells restore intrinsic homeostasis under metabolically toxic conditions by synergistically regulating multiple critical nodes, including glucolipid metabolism disorders, energy homeostasis imbalance, and inflammatory stress, thereby enhancing cell survival [30]. This suggests that the protective action of ginsenoside Rb1 against glucolipotoxicity does not rely on a single target but is achieved through the construction of a multidimensional homeostatic defense network.

### 3.3 Regulation of intestinal hormones and indirect protection

In addition to directly acting on the pancreatic islets, ginsenoside Rb1 exerts indirect protective effects by modulating systemic factors closely associated with islet function. For instance, ginsenoside Rb1 promotes the secretion of glucagon-like peptide-1 (GLP-1) from intestinal L cells, an effect associated with increased intracellular ATP/ADP ratio and calcium concentration, as well as upregulated proglucagon expression [31]. As a key incretin hormone, GLP-1 not only stimulates insulin secretion in a glucose-dependent manner but also inhibits β-cell apoptosis and promotes their proliferation. Therefore, enhancing GLP-1 secretion constitutes one of the peripheral pathways through which ginsenoside Rb1 exerts its islet-protective effects.

Studies have also suggested that ginsenoside Rb1 upregulates the expression of adiponectin [32], an adipokine with insulin-sensitizing and anti-inflammatory properties. Elevated adiponectin levels contribute to the amelioration of systemic and hepatic insulin resistance, thereby indirectly alleviating the long-term burden of compensatory insulin secretion on β-cells and creating a more favorable systemic metabolic microenvironment for them. This reveals another potential dimension of β-cell protection mediated by ginsenoside Rb1 through the “peripheral organ-pancreatic islet” crosstalk.

## 4 Multifaceted pharmacological effects of ginsenoside Rb1 and its therapeutic potential in diabetic complications

The protective effects and underlying mechanisms of ginsenoside Rb1 against diabetic complications have been extensively investigated in various preclinical models, as summarized in [Table 4]. This section mainly provides a concise narrative synthesis of the key findings across different complication types, highlighting common pathways and tissue-specific actions.

**Table 4:** Protective effects and mechanisms of ginsenoside Rb1 in diabetic complications.

Complication	Model	Key Findings	Mechanisms	Reference
Diabetic Nephropathy	High glucose-stimulated podocytes	Mitochondrial injury ↓, apoptosis ↓	Inhibition of aldose reductase activity	[33]
Diabetic Cardiomyopathy	*db/db* mice	Cardiac function improved, Ca^2+^ leakage ↓	RyR2 inhibition, SERCA2a activation	[34]
	Diabetic mice	Cardiac lipid accumulation ↓, inflammation ↓	Adipocytokine pathway activation	[35]
	*db/db* mice + chronic intermittent hypoxia	Oxidative stress ↓, cardiac function improved	AMPK/Nrf2/HO-1 activation	[18]
	*db/db* mice/cardiomyocytes	Cardiac hypertrophy ↓, lipid accumulation ↓	Mfn2 expression restored, mitochondrial fragmentation ↓	[36]
Diabetic Neuropathy	Schwann cells (intermittent high glucose)	Oxidative stress ↓, mitochondrial apoptosis ↓	Bax/Bcl-2 ratio ↓, ROS scavenging	[37]
Diabetic Retinopathy	STZ-induced diabetic rats	Retinal vascular permeability ↓, oxidative stress ↓	Nrf2 nuclear translocation ↑, GCLC/GCLM ↑	[14]
	High glucose-stimulated retinal endothelial cells	Cell viability ↑, mtDNA copy number ↑, ROS ↓	SIRT1/SIRT3 ↑, NAD-PARP-SIRT pathway modulation	[17]
Diabetic Wound Healing	Human diabetic fibroblasts (in vitro)	Proliferation ↑, collagen synthesis ↑	VEGF, TGF-β1, TIMP1 production ↑	[38]
Diabetic Atherosclerosis	Diabetic atherosclerosis model	Oxidative stress ↓, plaque progression ↓	Keap1 degradation, p47phox membrane translocation ↓, Nrf2/PGC-1α activation	[13]

*Abbreviation*: STZ: streptozotocin; HFD: high-fat diet; T2DM: type 2 diabetes mellitus; I/R: ischemia/reperfusion*.

↑ indicates increase/upregulation; ↓ indicates decrease/downregulation.

### 4.1 Ginsenoside Rb1 in diabetic kidney disease and related renal pathologies

A recent study investigated whether ginsenoside Rb1 counteracts high-dose erythropoietin-aggravated vascular calcification in the context of CKD. In adenine-induced CKD rats and β-glycerophosphate-treated vascular smooth muscle cells, ginsenoside Rb1 treatment attenuated calcification, restored smooth muscle marker expression, and inhibited Smad1/5/9 phosphorylation without affecting GATA6 or BMP2 expression. Molecular experiments demonstrated that ginsenoside Rb1 binds directly to BMP2, disrupting its receptor interaction and downstream signaling, thereby mitigating EPO-induced vascular toxicity. These findings have implications for diabetic kidney disease, given that CKD shares common pathological features with diabetic nephropathy [39]. In a separate investigation directly addressing diabetic kidney disease, He and colleagues revealed that ginsenoside Rb1 significantly alleviates high glucose-induced podocyte mitochondrial injury and apoptosis by directly binding to and inhibiting aldose reductase activity. This study provides experimental evidence elucidating the molecular mechanism underlying the therapeutic effect of *Panax notoginseng* in diabetic kidney disease, highlighting the potential of targeting the ginsenoside Rb1-aldose reductase interaction as a therapeutic strategy for mitigating diabetic nephropathy [33]. Collectively, these studies demonstrate that ginsenoside Rb1 exerts renoprotective effects through distinct molecular mechanisms—direct binding to BMP2 to attenuate vascular calcification in CKD and inhibition of aldose reductase to preserve podocyte function in diabetic conditions—underscoring its potential as a multi-target therapeutic agent for diabetic kidney disease and related renal pathologies.

### 4.2 Cardiovascular protective effects of ginsenoside Rb1

Ginsenoside Rb1 reduces myocardial infarct size and cell apoptosis in rat models of myocardial ischemia/reperfusion injury (MIRI) and protects H9C2 cells from hypoxia/reoxygenation-induced damage. The protective effect is mediated through inhibition of autophagy via activation of the PI3K/Akt/mTOR signaling pathway. These findings identify suppression of autophagy as a key mechanism by which ginsenoside Rb1 attenuates MIRI [40]. Ginsenoside Rb1 attenuates cardiac ischemia/reperfusion injury by inhibiting the burst of reactive oxygen species from mitochondrial complex I. It binds to the ND3 subunit, trapping complex I in a deactive conformation and reducing NADH dehydrogenase activity. This mechanism decreases mitochondrial ROS production, limits infarct size, and preserves cardiac function [15]. These findings indicate that Ginsenoside Rb1 confers cardioprotection against ischemia/reperfusion injury through dual mechanisms involving autophagy inhibition and mitochondrial complex I modulation. Beyond its role in acute ischemic events, emerging evidence has highlighted the therapeutic potential of ginsenoside Rb1 in diabetic cardiovascular disease, where chronic hyperglycemia and oxidative stress contribute to vascular complications.

A recent study demonstrated that ginsenoside Rb1 treatment for six weeks attenuated age-related structural remodeling and biomechanical dysfunction in the aorta of naturally aged mice. Ginsenoside Rb1 administration reduced cellular senescence markers (p21, SA-β-gal), decreased apoptotic cell death, and significantly lowered γ-H2AX expression, indicating reduced DNA damage. These findings suggest that Ginsenoside Rb1 delays vascular aging through mitigation of DNA damage, supporting its potential therapeutic application for age-associated vascular disorders [41]. A comprehensive review by Fan et al. systematically elucidated the pharmacological mechanisms through which ginsenosides intervene in metabolic syndrome and cardiovascular disease via multi-target modulation, providing an important theoretical basis for developing natural product-based therapeutic strategies for cardiometabolic diseases [42]. Furthermore, a study by Wang et al. demonstrated that ginsenoside Rb1 exerts a dual antioxidant effect in a diabetic atherosclerosis model through a synergistic mechanism involving direct binding to two distinct targets: Keap1 (promoting its SYVN1-mediated ubiquitin-dependent degradation) and p47phox (inhibiting its membrane translocation). This dual targeting consequently activates the Nrf2/PGC-1α signaling axis and suppresses NOX2 activity [13]. These findings collectively underscore the therapeutic potential of ginsenoside Rb1 in diabetic macrovascular complications by simultaneously enhancing endogenous antioxidant defenses and inhibiting pro-oxidant enzyme activation. Collectively, these studies demonstrate that ginsenoside Rb1 confers cardiovascular protection through multiple mechanisms, including attenuation of vascular aging via DNA damage reduction, broad multi-target modulation of metabolic and cardiovascular pathways, and specific dual antioxidant actions targeting the Keap1/Nrf2 and NOX2 axes in diabetic atherosclerosis.

Ginsenoside Rb1 has been shown to protect against diabetic cardiomyopathy through multiple mechanisms. In a mouse model of diabetic cardiomyopathy, ginsenoside Rb1 administration improved cardiac dysfunction by reducing Ca^2+^ leakage via inhibition of overactivated ryanodine receptor 2 (RyR2) and increasing Ca^2+^ uptake through sarcoplasmic reticulum Ca^2+^-ATPase 2a (SERCA2a), while also enhancing energy metabolism and reducing O-GlcNAcylation of calcium-handling proteins [34]. In a separate study, 12 weeks of ginsenoside Rb1 administration in diabetic mice reduced body weight, fat mass, hyperglycemia, hyperlipidemia, and insulin resistance, with associated decreases in cardiac lipid accumulation, inflammation, and oxidative stress leading to improved cardiac function via the adipocytokine pathway [35]. In *db/db* mice exposed to chronic intermittent hypoxia, ginsenoside Rb1 treatment for eight weeks improved glucose tolerance and cardiac function while reducing oxidative stress and myocardial collagen expression through activation of the AMPK/Nrf2/HO-1 signaling pathway [18]. Furthermore, in leptin receptor-deficient db/db mice and palmitic acid-treated cardiomyocytes, Ginsenoside Rb1 attenuated myocardial hypertrophy, diastolic dysfunction, and lipid accumulation by reversing diabetes-induced decreases in mitofusin 2 (Mfn2) expression and associated mitochondrial fragmentation [36]. These studies demonstrate that ginsenoside Rb1 exerts protective effects against diabetic cardiomyopathy through distinct but complementary mechanisms involving regulation of calcium homeostasis, systemic metabolic improvement, oxidative stress inhibition, and preservation of mitochondrial dynamics.

### 4.3 Diabetic neuropathy and neuroprotective effects of ginsenoside Rb1

A study investigating the neuroprotective properties of ginsenoside Rb1 demonstrated that it protected PC12 cells against Aβ25-35-induced cytotoxicity through activation of PPARγ and reduction of cellular cholesterol levels. Ginsenoside Rb1 treatment decreased reactive oxygen species production and lipid peroxidation while preserving cytoskeletal integrity and membrane surface rigidity, resulting in improved cell viability from approximately 50% in the AD model group to over 100% in the 50 μM ginsenoside Rb1 treatment group. These findings suggest that ginsenoside Rb1 may exert neuroprotective effects in Alzheimer's disease models through promoting reverse cholesterol transport and attenuating oxidative stress [43]. Additionally, Ginsenoside Rb1 inhibits mitochondrial complex I in astrocytes, reducing reactive oxygen species production and preventing their activation following ischemic insult. This facilitates the transfer of functional mitochondria from astrocytes to neurons, thereby restoring neuronal mitochondrial membrane potential and oxygen consumption rate. The neuroprotective effect of ginsenoside Rb1 in ischemic brain injury is mediated through astrocyte mitochondrial transfer, highlighting astrocytes as a key therapeutic target [16]. These findings collectively indicate that ginsenoside Rb1 confers neuroprotection through distinct yet complementary mechanisms, including the modulation of cholesterol metabolism and the facilitation of intercellular mitochondrial transfer.

A separate investigation by Yang et al. examined total saponins extracted from steam-processed American ginseng, reporting that the AGTS5 fraction conferred neuroprotection in zebrafish larvae with AlCl_3_-induced neuronal injury and adult zebrafish with cognitive impairment. This protective effect coincided with the transformation of primary ginsenosides into rare ginsenosides during steaming and operated through mitigation of oxidative damage, neuroinflammatory responses, and cholinergic dysfunction. These observations support the potential application of processed American ginseng extracts, particularly AGTS5, as functional food ingredients for cognitive enhancement [44]. In the context of diabetic neuropathy, Xue and colleagues reported that ginsenoside Rb1 attenuated intermittent high glucose-induced oxidative stress and mitochondrial apoptosis in Schwann cells through downregulation of the Bax/Bcl-2 ratio and reactive oxygen species scavenging, with effects exhibiting concentration dependence [37]. These findings indicate that ginsenoside Rb1 may preserve Schwann cell function and survival under fluctuating glycemic conditions, suggesting therapeutic potential for diabetic peripheral neuropathy. Collectively, these studies demonstrate that ginsenoside Rb1 and related ginseng-derived compounds exert neuroprotective effects across multiple model systems—including Alzheimer's disease, chemical-induced cognitive impairment, and diabetic neuropathy—through mechanisms involving PPARγ activation, cholesterol regulation, oxidative stress attenuation, and preservation of mitochondrial function.

### 4.4 Ginsenoside Rb1 in diabetic retinopathy: antioxidant and mitochondrial protective mechanisms

In vivo studies have demonstrated the protective effects of ginsenoside Rb1 against diabetic retinopathy. In streptozotocin-induced diabetic rats, four weeks of ginsenoside Rb1 administration (20 or 40 mg/kg/day) attenuated diabetes-induced increases in retinal blood vessel diameter and vascular permeability, while partially inhibiting oxidative stress markers by reducing malondialdehyde content and preventing glutathione depletion. These improvements were associated with increased nuclear translocation of Nrf2 and upregulation of its target genes GCLC and GCLM, suggesting that ginsenoside Rb1 attenuates diabetic retinopathy through enhancement of retinal antioxidant capacity [14]. Complementing these in vivo findings, cellular investigations using rat retinal capillary endothelial cells exposed to high glucose conditions revealed that ginsenoside Rb1 treatment increased cell viability and mitochondrial DNA copy number, reduced reactive oxygen species generation, and restored cellular redox balance by modulating SOD, CAT, NOX, and PARP activities. Mechanistically, ginsenoside Rb1 enhanced SIRT activity and SIRT1/SIRT3 expression while targeting the NAD-PARP-SIRT signaling pathway, indicating that Ginsenoside Rb1 protects retinal endothelial cells from high glucose-induced damage through regulation of the cellular redox state and NAD-dependent signaling [17]. A comprehensive review by Ren et al. systematically elucidated that natural bioactive constituents, exemplified by ginsenoside Rb1, can synergistically restore blood-retinal barrier dysfunction through multi-target regulation of the oxidative stress-inflammation-programmed cell death network, thereby offering a novel multi-pathway intervention strategy for drug development in retinal diseases [45]. Collectively, these in vivo, cellular, and mechanistic studies provide converging evidence that ginsenoside Rb1 exerts protective effects against diabetic retinopathy through coordinated enhancement of antioxidant defenses, preservation of mitochondrial function, and modulation of NAD-dependent signaling pathways, supporting its potential as a multi-target therapeutic agent for this complication.

### 4.5 In vitro effects on diabetic fibroblast function

An in vitro study demonstrated that ginsenoside Rb1 (10 ng/mL) treatment of human diabetic fibroblasts significantly increased cell proliferation, collagen synthesis, and the production of vascular endothelial growth factor, transforming growth factor-β1, and tissue inhibitor of metalloproteinases 1 compared to untreated controls. No significant differences were observed in basic fibroblast growth factor or matrix metalloproteinase 1 levels [38]. These findings suggest that *Panax ginseng* extract containing Ginsenoside Rb1 may promote diabetic wound healing through enhancing fibroblast function and modulating extracellular matrix metabolism.

### 4.6 Antidepressant effects of ginsenoside Rb1: modulation of neuroinflammation and synaptic plasticity

Ginsenoside Rb1 inhibited LPS-induced inflammation and NF-κB pathway activation in astrocytes and suppressed LPS and ATP-induced astrocytic pyroptosis. The protective effect was associated with enhanced mitophagy and reduced reactive oxygen species production, as inhibition of mitophagy by cyclosporin A reversed these effects. In a chronic unpredictable mild stress rat model, Ginsenoside Rb1 administration improved depressive-like behaviors, inhibited astrocytic pyroptosis, and preserved synaptic structure and density, with efficacy comparable to escitalopram [22]. Ginsenoside Rb1 treatment in a chronic unpredictable mild stress mouse model downregulated hippocampal miR-134, which directly targets the BDNF 3′UTR. Hippocampus-specific overexpression of miR-134 abolished the antidepressant-like effects of Ginsenoside Rb1, including the restoration of dendritic spine density, synaptic ultrastructure, long-term potentiation, and BDNF-TrkB signaling. These findings indicate that Ginsenoside Rb1 ameliorates stress-induced depression-like behaviors by modulating synaptic plasticity through the miR-134/BDNF pathway [46]. In a lipopolysaccharide-induced neuroinflammation mouse model, ginsenoside Rb1 ameliorated depressive-like behavior through inhibition of the MAPK/NF-κB signaling pathway. Ginsenoside Rb1 and its metabolite F2 were identified as active compounds, with F2 exhibiting greater antidepressant potency. Mechanistically, ginsenoside Rb1 suppressed peripheral and hippocampal inflammation, restored glucocorticoid receptor function, reduced indoleamine 2,3-dioxygenase activity, and increased serotonin levels and 5-HT1A receptor expression [24]. In a chronic restraint stress mouse model, ginsenoside Rb1 reversed depressive-like behaviors by inhibiting TLR4/NF-κB/C3 signaling in hippocampal astrocytes. This suppression of astrocytic complement C3 production reduced microglial number and promoted their morphological transition from an amoeboid pro-inflammatory phenotype to a ramified anti-inflammatory phenotype. Consequently, Ginsenoside Rb1 attenuated microglia-mediated synaptic elimination, as evidenced by preserved synaptophysin and PSD95 levels and maintained dendritic spine density [23]. These studies demonstrate that ginsenoside Rb1 exerts antidepressant effects through convergent mechanisms involving suppression of astrocytic and microglial inflammation, regulation of the miR-134/BDNF axis, and preservation of synaptic structure and function. Given that diabetes mellitus is associated with an increased risk of depression—a comorbidity often linked to chronic neuroinflammation and impaired BDNF signaling—the multifaceted neuroprotective profile of ginsenoside Rb1 suggests its potential application in managing diabetes-associated depressive symptoms.

### 4.7 Ginsenoside Rb1 in obesity

In high-fat diet-induced obese mice, chronic ginsenoside Rb1 treatment improved central leptin sensitivity and restored leptin-JAK2-STAT3 signaling, leading to enhanced leptin-induced BDNF expression in the prefrontal cortex. In cultured prefrontal cortical neurons, Rb1 prevented palmitic acid-induced impairment of leptin-stimulated BDNF expression, neurite outgrowth, and synaptogenesis [47]. Separately, four weeks of Rb1 administration (40 mg/kg/day) in high-fat diet-induced obese mice reduced body weight and adipocyte size, improved glucose tolerance, and increased basal metabolic activity. Mechanistically, Ginsenoside Rb1 downregulated myostatin (MSTN) expression in adipose tissue and differentiated C2C12 and 3T3-L1 cells while upregulating fibronectin type III domain-containing 5 (FNDC5) expression; MSTN overexpression attenuated ginsenoside Rb1-mediated reduction of lipid droplet accumulation in adipocytes [48]. These findings indicate that ginsenoside Rb1 exerts anti-obesity effects through distinct but complementary mechanisms involving restoration of central leptin sensitivity and regulation of the MSTN/FNDC5 signaling pathway.

## 5 Summary of potential signaling pathways

To better integrate the diverse signaling pathways reported for ginsenoside Rb1, we have categorized the underlying mechanisms into primary (direct target binding) and secondary or context-dependent effects. Primary mechanisms refer to those supported by experimental evidence of direct physical interaction between ginsenoside Rb1 and a specific protein, as validated by methods such as surface plasmon resonance (SPR), cellular thermal shift assay (CETSA), isothermal titration calorimetry (ITC), or molecular docking with functional validation. In contrast, secondary or context-dependent effects include downstream signaling events (e.g., activation of AMPK, inhibition of NF-κB, nuclear translocation of Nrf2) and systemic responses (e.g., gut microbiota remodeling, changes in metabolite profiles) that occur indirectly following target engagement or under specific pathological conditions [Table 5]. summarizes the currently validated direct molecular targets of ginsenoside Rb1 along with their experimental evidence, while listing representative indirect pathways that are frequently described in the literature. This classification helps readers distinguish established direct interactions from downstream events that may vary depending on cell type, disease model, or experimental context. The validated direct molecular targets of ginsenoside Rb1 are summarized in [Table 5], along with representative indirect mechanisms that operate downstream or in a context-dependent manner.

**Table 5:** Classification of primary and secondary mechanisms of ginsenoside Rb1.

A. Direct molecular targets			
Target	Validation method(s)	Regulatory outcome	Reference
TLR4	SPR, CETSA	Inhibits receptor dimerization, blocks MyD88-NF-κB/MAPK	[21]
PPARγ	Molecular docking, antagonist assay	Activates transcription, suppresses NF-κB	[7]
BMP2	SPR	Inhibits BMP2-receptor interaction, suppresses Smad1/5/9	[39]
Aldose reductase	Enzyme kinetics, molecular docking	Inhibits enzymatic activity	[33]
Keap1	Co-IP, molecular docking, ubiquitination assay	Promotes SYVN1-mediated ubiquitin-dependent degradation	[13]
p47phox	Co-IP, molecular docking	Inhibits membrane translocation, suppresses NOX2 activity	[13]

B. Representative indirect/context-dependent mechanisms		
Pathway/effect	Description	Reference
AMPK activation	Enhanced phosphorylation of AMPK, often downstream of SIRT1 or upstream of Nrf2	[12,40]
NF-κB inhibition	Suppression of p65 nuclear translocation, decreased pro-inflammatory cytokines	[7,21]
Nrf2 nuclear translocation	Increased Nrf2 activity leading to antioxidant gene expression (HO-1, GCLC, GCLM)	[13,14]
Gut microbiota remodeling	Altered composition (e.g., increased *Lactobacillus*, *Bacteroides*), changed SCFA/FFAR4 signaling	[8,10,11]

**Fig. 3:**
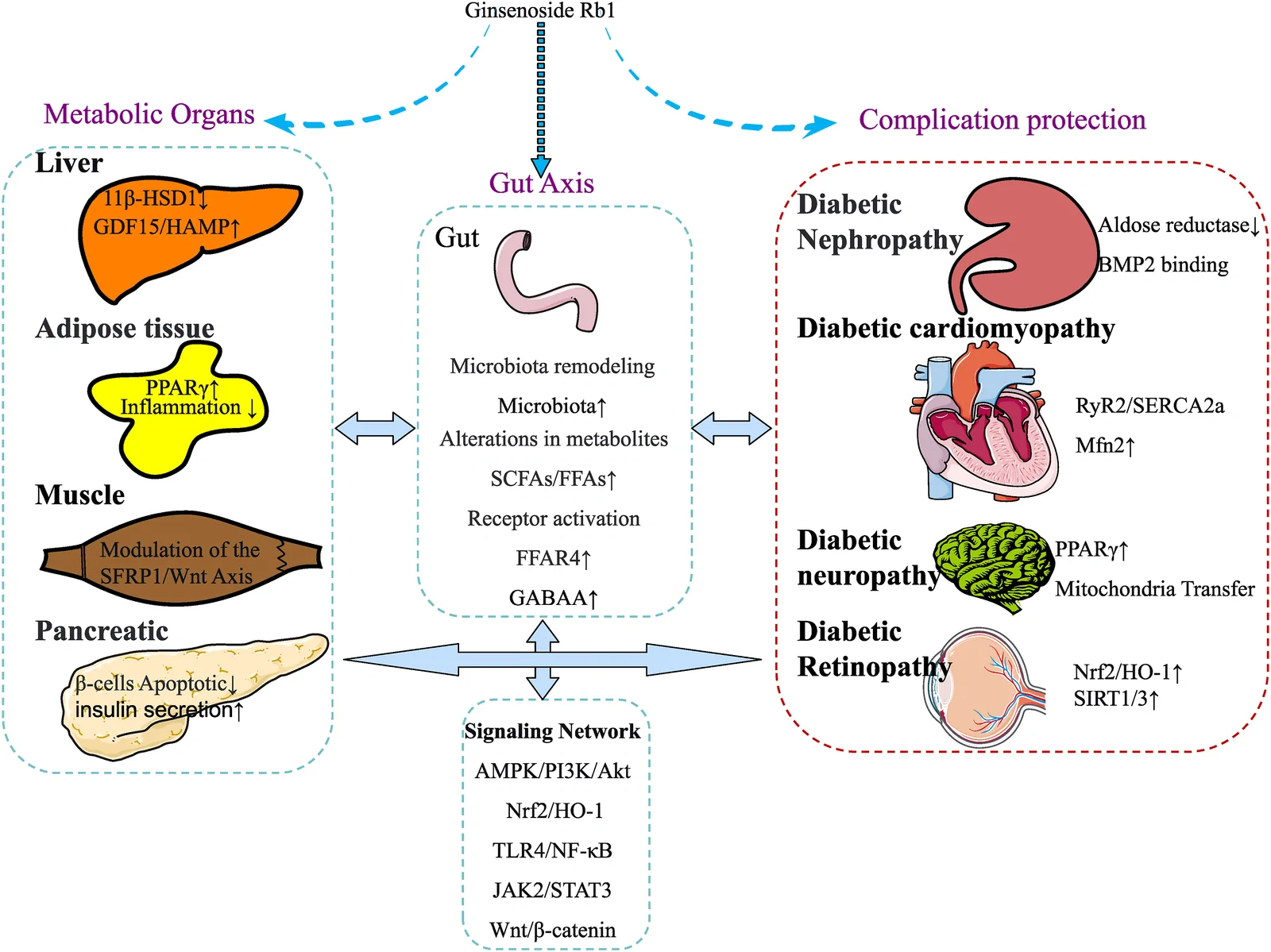
Multi-target mechanisms of Ginsenoside Rb1 in diabetes. Abbreviation: AMPK, AMP-activated protein kinase; eNOS, endothelial nitric oxide synthase; GCLC, glutamate-cysteine ligase catalytic subunit; GCLM, glutamate-cysteine ligase modifier subunit; HO-1, heme oxygenase 1; Keap1, Kelch-like ECH-associated protein 1; NAD+/NADH, nicotinamide adenine dinucleotide (oxidized/reduced); NOX2, NADPH oxidase 2; Nrf2, nuclear factor erythroid 2-related factor 2; p47phox, a cytosolic subunit of NADPH oxidase; PGC-1α, peroxisome proliferator-activated receptor gamma coactivator 1-alpha; SIRT, sirtuin; SYVN1, synoviolin 1 (an E3 ubiquitin ligase).

[Fig. 3] Schematic overview of the pleiotropic protective effects of Ginsenoside Rb1 across multiple organs and signaling networks. Ginsenoside Rb1 exerts its therapeutic effects through coordinated modulation of metabolic organs, the gut axis, and intracellular signaling pathways, ultimately providing protection against diabetic complications.

The therapeutic effects of ginsenoside Rb1 in diabetes and its complications are mediated through modulation of multiple interconnected signaling networks across different tissues and pathological contexts. In the context of insulin resistance and metabolic dysregulation, Ginsenoside Rb1 enhances peripheral insulin sensitivity through suppression of 11β-HSD1 in liver and adipose tissue [4], modulation of the SFRP1/Wnt/β-catenin axis in skeletal muscle [5], synergistic regulation of hepatic GDF15/hepcidin signaling [6], and PPARγ-mediated attenuation of adipose tissue inflammation [7]. Concurrently, ginsenoside Rb1 exerts gut microbiota-dependent effects through remodeling microbial composition and activating the FFAR4 receptor via altered fatty acid metabolite profiles, establishing a “microbiota-metabolite-receptor” regulatory axis that improves systemic glucose and lipid metabolism despite low oral bioavailability [[8], [9], [10]]. At the cellular level, ginsenoside Rb1 activates antioxidant defense mechanisms through AMPK/SIRT1/Nrf2 signaling pathways, enhancing expression of antioxidant enzymes and restoring redox balance [11,12,19]. Its anti-inflammatory effects are mediated through direct binding to TLR4, inhibiting receptor dimerization and subsequent MyD88-NF-κB/MAPK signaling cascades [20,21,25]. In pancreatic β-cells, Ginsenoside Rb1 promotes insulin secretion through PKA/cAMP and PI3K/Akt pathway activation [26] and enhances GLP-1 secretion from intestinal L cells [27], while protecting against glucolipotoxicity-induced apoptosis through downregulation of Bax/Bax/caspase-3 and modulation of cellular stress networks [28,29]. In diabetic complications, Ginsenoside Rb1 activates tissue-specific protective signaling pathways. In diabetic kidney disease, Ginsenoside Rb1 targets aldose reductase to preserve podocyte function [39] and binds directly to BMP2 to attenuate vascular calcification [32]. In cardiovascular complications, Ginsenoside Rb1 activates the Nrf2/PGC-1α axis while suppressing NOX2 activity through dual targeting of Keap1 and p47phox [15], and attenuates vascular aging through reduction of DNA damage [33]. In diabetic neuropathy, Ginsenoside Rb1 activates PPARγ and preserves mitochondrial function [13,41], while in diabetic retinopathy, it enhances Nrf2 nuclear translocation and NAD-PARP-SIRT signaling to protect retinal endothelial cells [18,34,35]. A study by Guo et al. systematically revealed that a group of natural products, represented by ginsenoside Rb1, can reshape NAD metabolic homeostasis through synergistic regulation of core signaling networks, including the NAMPT/NAD/SIRT and AMPK/Nrf2 pathways, via multidimensional pharmacological interventions. This provides a new paradigm for natural product research that offers both efficacy and safety in the targeted treatment of metabolic diseases [49]. Collectively, these findings underscore that the therapeutic potential of ginsenoside Rb1 in metabolic disorders stems from its ability to modulate interconnected signaling networks governing energy metabolism, oxidative stress defense, mitochondrial homeostasis, and inflammatory responses across multiple tissues, thereby addressing the complex pathophysiology of diabetes and its complications through integrated multi-target mechanisms rather than isolated linear pathways.

## 6 Pharmacokinetics and safety considerations

[Table 6] summarizes the pharmacokinetic parameters and bioavailability of Ginsenoside Rb1, as reported in various studies. The data encompass absorption, distribution, metabolism, and excretion (ADME) characteristics evaluated in different experimental models, primarily rats and humans.

**Table 6:** Pharmacokinetic parameters and bioavailability of Ginsenoside Rb1.

Parameter	Value/Observation	Model/Species	Notes	Reference
Oral Bioavailability	∼4.35%	Rat	Fits a two-compartment model	[50]
Absolute Bioavailability	4.35% (Rb1), 18.40% (Rg1)	Rat	*Panax notoginseng* saponins	[50]
Major Metabolites	Rd, Compound K, F2, Rg3, Rk1+Rg5, Gypenoside XVII	Rat/Human	Produced by gut microbiota-mediated metabolism	[25,51,52]
Tmax (Rb1)	Variable	Rat	Influenced by gut microbiota composition	[51]
Tmax (Compound K)	Shortened	Healthy male subjects	Bioconverted red ginseng extract vs. conventional red ginseng	[52]
Cmax (Rg3, Rk1+Rg5, F2, Compound K)	Increased	Healthy male subjects	Bioconverted red ginseng extract	[52]
Gut Microbiota Influence	Correlated with *Bacteroides* and glycoside hydrolase (GH2, GH92, GH20) abundance	Rat	Bidirectional relationship: Rb1 alters microbiota, microbiota affects Rb1 metabolism	[51]
Tissue Distribution	Widely distributed in various organs	Animal studies	Can cross blood-brain barrier (low but effective)	[11,53]
Elimination Half-Life	Species-dependent	Rat/Dog	Requires further investigation	[53]
Bioavailability Enhancement	Higher systemic exposure	Human	Bioconversion increases absorption rate of bioactive ginsenosides	[52]
Metabolic Conversion	100% molar yield (Rb1 → Gypenoside XVII)	*In vitro* (recombinant β-glucosidase)	Enzymatic production by *Bifidobacterium dentium*-derived enzyme	[25]

Abbreviations: Tmax, time to reach maximum concentration; Cmax, maximum plasma concentration; Compound K (CK), 20-O-β-D-glucopyranosyl-20(S)-protopanaxadiol; GH, glycoside hydrolase family.

### 6.1 In vivo fate of ginsenoside Rb1

A study investigated the impact of a 30-day ginsenoside Rb1 intervention on gut microbiota composition and Ginsenoside Rb1 pharmacokinetics in rats. Oral administration of Rb1 significantly altered the systemic exposure and metabolic clearance rate of both Ginsenoside Rb1 and its metabolite Rd, while increasing the relative abundance of *Bacteroides cellulosilyticus* and specific glycoside hydrolase families (GH2, GH92, GH20). Correlation analysis revealed associations between Ginsenoside Rb1 pharmacokinetic parameters and the abundance of specific Bacteroides species and glycoside hydrolase families, indicating a bidirectional relationship between Ginsenoside Rb1 administration and gut microbiota composition [51]. A study by Xu et al. first elucidated that ginsenosides Rb1 and Rg1 derived from *Panax notoginseng* saponins exhibit pharmacokinetic characteristics conforming to a two-compartment model in rats, with absolute bioavailability values of 4.35% and 18.40%, respectively. This research provided a crucial theoretical basis for optimizing formulations to improve the oral bioavailability of *Panax notoginseng* saponins [50]. A randomized crossover study investigated the pharmacokinetic profiles of multiple ginsenosides following single oral administration of red ginseng and bioconverted red ginseng extract in 14 healthy Korean men. Bioconverted red ginseng extract demonstrated higher maximum plasma concentration and area under the curve for ginsenosides Rg3, Rk1+Rg5, F2, and compound K, as well as shorter time to maximum concentration for compound K, compared to standard red ginseng. These findings indicate that bioconversion enhances the systemic exposure and absorption rate of bioactive ginsenosides, providing important pharmacokinetic data for optimizing ginseng-based therapeutic applications [52]. A comprehensive review by Ling et al. systematically elaborated on the therapeutic potential of ginsenoside Rb1 in neurological disorders through its multi-target neuroprotective mechanisms involving anti-inflammatory, antioxidant, anti-apoptotic, and anti-autophagic effects, while also providing a thorough summary of research progress on its pharmacokinetic properties and mechanisms of action [53]. Collectively, these findings highlight the inherent challenge of low oral bioavailability of ginsenoside Rb1, underscoring the need for advanced formulation strategies or structural modifications to enhance its therapeutic applicability while maintaining its multi-target pharmacological profile.

### 6.2 Preclinical and clinical safety

A study by Sharma et al. systematically elaborated that ginsenoside Compound K, as a biotransformation product of high-polarity ginsenosides such as Ginsenoside Rb1, overcomes bioavailability limitations through innovative formulation strategies including nanocarriers, exhibiting multi-target pharmacological activities and favorable safety profiles, thereby providing critical research directions for advancing the clinical translation of this candidate therapeutic agent [54]. These findings collectively suggest that while ginsenoside Rb1 itself demonstrates promising preclinical safety, strategic optimization of its pharmacokinetic properties—through approaches such as harnessing its bioactive metabolites or employing advanced drug delivery systems—represents a viable pathway for developing safe and effective ginsenoside Rb1-based therapeutics for clinical application.

## 7 Limitations of current research and future perspectives

Current investigations into ginsenoside Rb1 for diabetes management remain predominantly confined to cellular and animal models, with a conspicuous absence of large-scale, high-quality randomized controlled trials in human populations. A significant challenge impeding its clinical translation is the compound's complex in vivo metabolic profile and inherently low oral bioavailability. Furthermore, the synergistic mechanisms underlying its multi-component, multi-target actions require more comprehensive elucidation.

Future research endeavors should prioritize the following directions: First, well-designed clinical trials are urgently needed to rigorously evaluate the efficacy and safety of ginsenoside Rb1 in patients with diabetes. Second, the development of advanced drug delivery systems, such as nanoformulations, holds promise for substantially enhancing its bioavailability. Third, deeper mechanistic exploration of its “gut-axis” mediated regulation of glucose metabolism represents a particularly compelling avenue. Fourth, the potential for combination therapies integrating Ginsenoside Rb1 with existing antidiabetic agents warrants systematic investigation to identify possible synergistic benefits. Addressing these critical gaps will be essential for translating the promising preclinical findings of ginsenoside Rb1 into clinically viable therapeutic applications.

## 8 Conclusion

Ginsenoside Rb1 demonstrates significant therapeutic potential in diabetes management through multi-target mechanisms, including enhancing insulin sensitivity via AMPK/PI3K/Akt signaling, modulating the gut microbiota-SCFA metabolic axis, and protecting pancreatic β-cells by inhibiting ER stress and mitochondrial apoptosis. In diabetic complications, ginsenoside Rb1 exerts protective effects against nephropathy, cardiovascular disease, neuropathy, and retinopathy through regulation of TGF-β1/Smad, Nrf2/HO-1, and GDF15/HAMP pathways. Despite these promising findings, critical challenges remain, particularly its low oral bioavailability (approximately 4.35%) and the paucity of large-scale clinical trials. The low oral bioavailability of ginsenoside Rb1 (approximately 4.35%) is primarily attributed to its poor intestinal permeability, extensive gut microbiota-mediated deglycosylation, and hepatic first-pass metabolism. Future research should prioritize advanced formulation strategies to enhance bioavailability, rigorous clinical validation, and elucidation of synergistic mechanisms with existing antidiabetic agents. Collectively, current evidence establishes ginsenoside Rb1 as a valuable multi-target lead compound for diabetes therapeutics, providing a robust foundation for its clinical translation.

## Declaration of competing interest

The authors declare that they have no known competing financial interests or personal relationships that could have appeared to influence the work reported in this paper.
